# Combined approaches to rescue an entrapped pancreatic stone basket

**DOI:** 10.1055/a-2729-2731

**Published:** 2025-11-19

**Authors:** Fan Fan, Jin-Hui Yi, Aiqiao Fang, Liang-Hao Hu

**Affiliations:** 1 71185 Department of Gastroenterology, Peking University People’s Hospital, Beijing, China; 2 481107 School of Medicine, NanKai University, Tianjin, China; 3 651943 Department of Gastroenterology, The First Medical Center of Chinese PLA General Hospital, Beijing, China; 4 12520 Digestive Endoscopy Center, Changhai Hospital, Naval Medical University, Shanghai, China; 5 12520 Department of Gastroenterology, Shanghai Changhai Hospital, Naval Medical University, Shanghai, China

A 56-year-old male patient with chronic pancreatitis presented to our hospital. Computed tomography identified multiple radiopaque calculi within the pancreatic duct ( Fig. 1 ). The patient underwent three sessions of pancreatic extracorporeal shock wave lithotripsy (P-ESWL) followed by endoscopic retrograde cholangiopancreatography (ERCP) for stone extraction 1 2 .

**Fig. 1:**
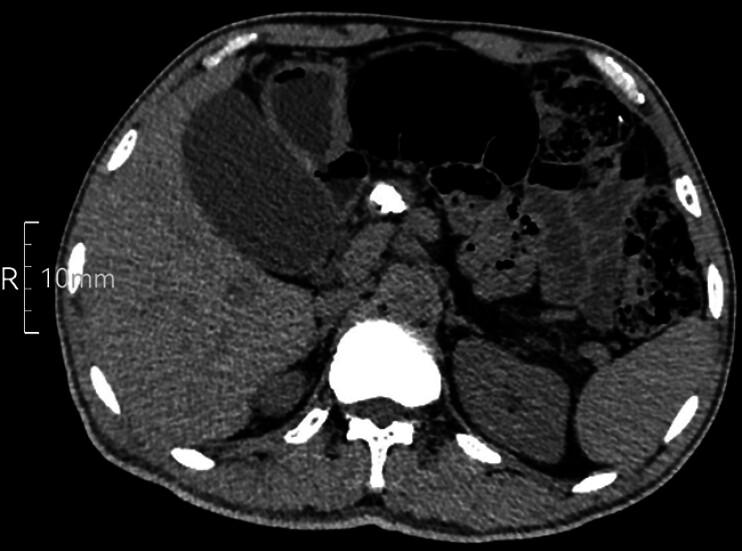
Computed tomography identified multiple radiopaque calculi within the pancreatic duct.

ERCP revealed main pancreatic duct stenosis with multiple filling defects. Sphincterotomy and subsequent balloon dilation were performed, achieving ductal expansion to 1 cm in diameter. A spiral stone retrieval basket was employed to extract the stones. However, the basket became entrapped in the pancreatic duct due to an excessive amount of adherent stone debris ( Fig. 2 ). The handle of the basket was cut ( Fig. 3 **a** ), and the duodenoscope was withdrawn from the alimentary tract with a basket wire inserted into the working channel simultaneously, leaving the stone-laden basket within the pancreas and basket wire extending out of the patient’s mouth ( Fig. 3 **b–d** ). An additional session of P-ESWL was subsequently performed.

**Fig. 2:**
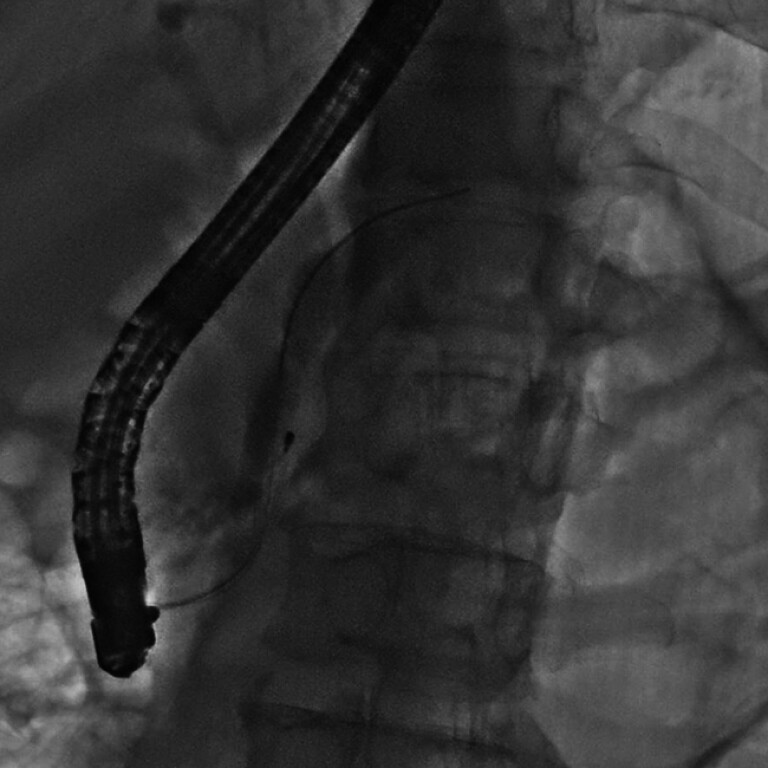
The spiral stone retrieval basket was entrapped in the pancreatic duct.

**Fig. 3:**
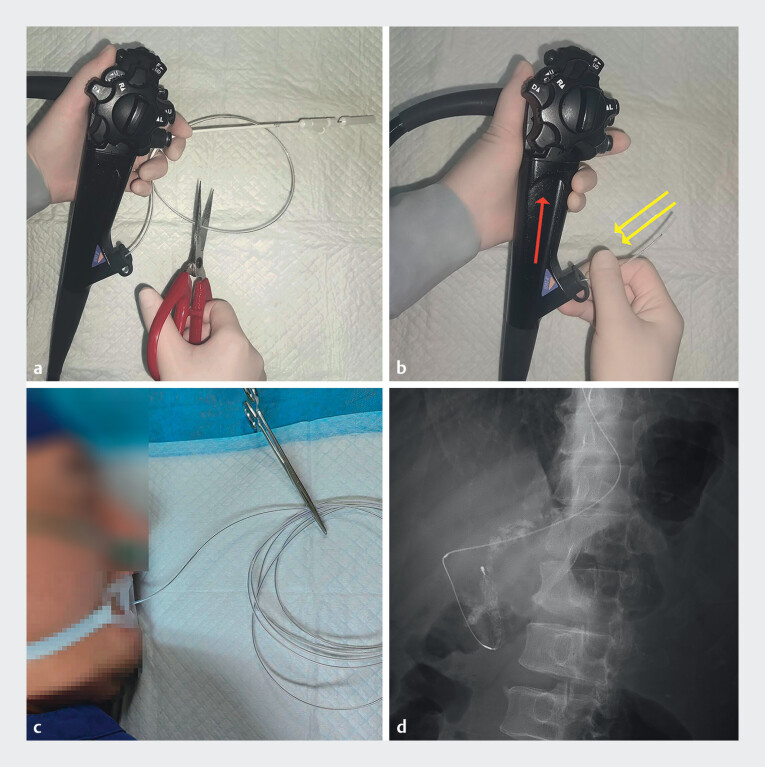
**a** The handle of the spiral stone retrieval basket was cut. **b** The duodenoscope was withdrawn from the alimentary tract while the basket wire inserted into the working channel simultaneously. **c** The basket wire extended out of the patient’s mouth. **d** The stone-laden basket within the pancreas. The red arrows show the movement of the duodenoscope and the yellow arrows show the movement of the basket wire.

Forceps was then introduced through the working channel of the duodenoscope to grasp the end of the basket wire ( Fig. 4 **a, b** ). The forceps, along with the wire, were then retracted through the working channel while the scope was re-advanced to the duodenal papilla ( Fig. 4 **c** and **d** ). Attempts were made to extract the basket, but significant resistance was encountered. A balloon catheter was inserted to further dilate the papilla to a diameter of 1.1 cm ( Fig. 5 **a** ). Despite this, basket retrieval remained challenging. A sphincterotome was then utilized to fragment the stone debris within the basket, reducing the overall volume of the stone–basket complex ( Fig. 5 **b, c** ). Ultimately, the basket, along with the stones, was successfully extracted ( Fig. 5 **d** , Video 1 ).

**Fig. 4:**
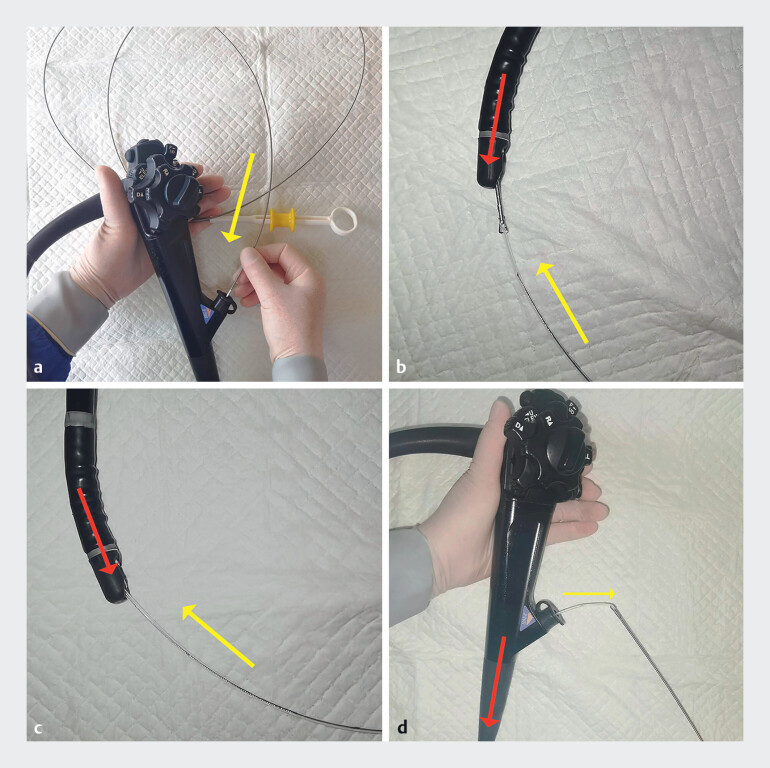
**a** Forceps was introduced through the working channel of the duodenoscope. **b** Forceps grasped the end of the basket wire. **c** The forceps, along with the wire, were retracted through the working channel while duodenoscope re-advanced. **d** The basket wire was retracted through the working channel. The red arrows show the movement of the duodenoscope and the yellow arrows show the movement of the basket wire and forceps.

**Fig. 5:**
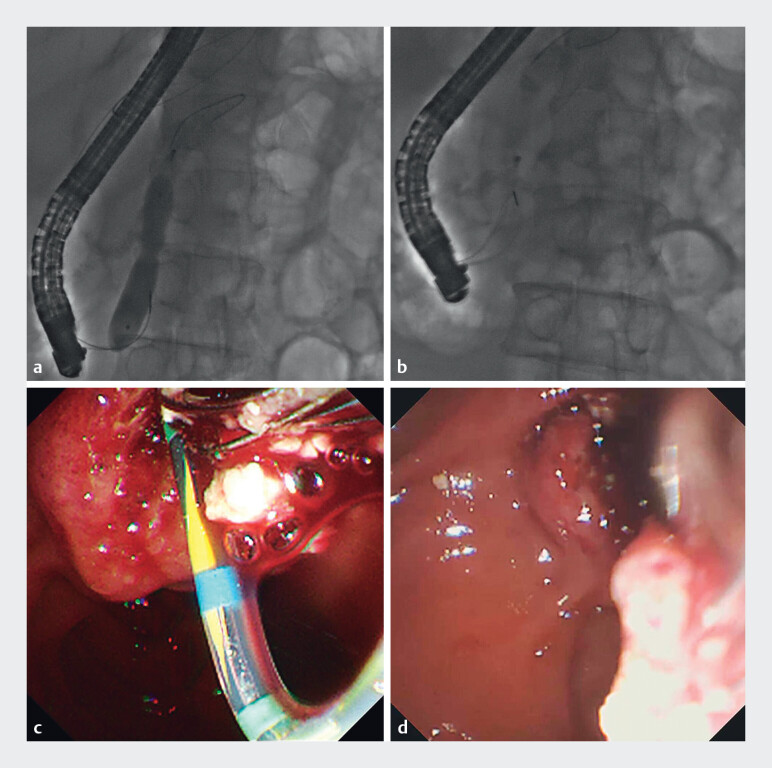
**a** A balloon catheter was inserted to further dilate the papilla. **b** Basket retrieval remained challenging. **c** A sphincterotome was utilized to fragment the stone debris within the basket. **d** The basket, along with the stones, were successfully extracted.

Sphincterotome-assisted fragmentation to rescue an entrapped pancreatic stone basket.Video 1

Basket entrapment is an undesirable complication during endoscopic pancreatic stone removal. Various rescue techniques, including P-ESWL, sphincterotomy, and laser lithotripsy, have been reported in the literature to cope with such situations 3 4 5 . In this case, we successfully managed this complicated scenario through a combination of approaches, including P-ESWL, balloon dilation and an innovative sphincterotome-assisted fragmentation technique.

Endoscopy_UCTN_Code_CPL_1AK_2AC

## Notes

Endoscopy E-Videos https://eref.thieme.de/e-videos
*E-Videos* is an open access online section of the journal *Endoscopy* , reporting on interesting cases and new techniques in gastroenterological endoscopy. All papers include a high-quality video and are published with a Creative Commons CC-BY license. Endoscopy E-Videos qualify for HINARI discounts and waivers and eligibility is automatically checked during the submission process. We grant 100% waivers to articles whose corresponding authors are based in Group A countries and 50% waivers to those who are based in Group B countries as classified by Research4Life (see: https://www.research4life.org/access/eligibility/ ). This section has its own submission website at https://mc.manuscriptcentral.com/e-videos .
